# Advances in Research on Brain Health and Dementia: Prevention and Early Detection of Cognitive Decline and Dementia

**DOI:** 10.3390/brainsci14040353

**Published:** 2024-04-01

**Authors:** Takao Yamasaki, Takuro Ikeda

**Affiliations:** 1 Department of Neurology, Minkodo Minohara Hospital, Fukuoka 811-2402, Japan; 2 Kumagai Institute of Health Policy, Fukuoka 816-0812, Japan; 3 Department of Physical Therapy, Faculty of Medical Sciences, Fukuoka International University of Health and Welfare, Fukuoka 814-0001, Japan; ikeda@takagigakuen.ac.jp

## 1. Introduction

Although “brain health” has many definitions, the core definition is the maintenance of optimal brain structure and function [1]. As the world’s population ages rapidly, brain health is seriously threatened by various types of dementia and high-risk conditions [2]. The main types of dementia are Alzheimer’s disease (AD), dementia with Lewy bodies (DLB), and vascular dementia [3]. Mild cognitive impairment (MCI), subjective cognitive decline (SCD), cognitive frailty, and motoric cognitive risk syndrome (MCR) are high-risk conditions for dementia [3,4,5,6]. These conditions place a significant burden not only on the patient but also on their families and caregivers and greatly increase social costs, such as medical and nursing care costs [2]. Therefore, countermeasures against these conditions have become an important issue worldwide, and early detection and intervention are critical among these measures. This Special Issue brings together research from various fields focused on maintaining brain health and the early detection and intervention of dementia and its precursor conditions.

## 2. Special Issue Overview

### 2.1. Early Detection of the AD Continuum

The most common type of dementia is AD, accounting for 60–80% of all cases [3]. AD forms a continuum spanning three stages: preclinical AD, MCI due to AD, and AD dementia [3]. Advanced preclinical AD is known as SCD [4]. In these continua, patients at earlier stages of MCI due to AD are better targets for early detection and intervention [2]. To detect the early AD continuum, typical cognitive function screening tests for community-dwelling elderly individuals include the Mini-Mental State Examination (MMSE) and Montreal Cognitive Assessment (MoCA) [7,8].

This Special Issue includes two research papers on additional tests that increase the sensitivity of existing ones (e.g., MMSE and MoCA). Trapp et al. (Contribution 1) developed a cognitive screening method using Lego^®^ Duplo^®^ building blocks to test the assembly of simple animal shapes. This test targets various cognitive functions such as spatial awareness, hand–eye coordination, working memory, and executive function. Patients with mild dementia and MCI performed worse on assembly tasks than controls, suggesting that adding this simple and quick test to existing tests significantly increases the sensitivity of dementia and MCI detection. Impaired odor discrimination is a useful early marker of MCI and dementia [9]. Trapp et al. (Contribution 2) used the Sniffin’ Sticks odor identification test as a standardized test and a brief odor identification test requiring the identification of coffee scents. Patients with dementia and MCI exhibited impaired odor discrimination in both tests. Therefore, adding a brief odor identification test to existing cognitive screening tests may significantly increase the sensitivity of dementia and MCI detection.

Recent advances in digital technology have increased the number of screening methods using mobile devices, desktops, and the Web. This increase has further accelerated, especially owing to the COVID-19 pandemic [7]. Screening tests that use digital technology have advantages over paper-and-pencil tests, such as being more objective and quantifiable, requiring no expert evaluation, and faster testing [7,8]. Thus, it is very useful for identifying potential patients among community-dwelling elderly people. This Special Issue includes studies that examine the use of digital technology. Wang et al. (Contribution 3) developed a two-minute test focused on the ability to perform spatial navigation tasks. In this test, the subjects must use their fingers to manipulate virtual steering on a tablet to control the ball and eliminate all target cubes. The authors demonstrated that this test could detect patients with MCI due to AD with the advantages of high accuracy, speed, low cost, alert efficiency, and intelligence. Therefore, this test is considered to have a high application value in community screening.

Representative neuroimaging biomarkers of AD include magnetic resonance imaging (MRI), positron emission tomography, near-infrared spectroscopy (NIRS), and electroencephalography [10,11]. This Special Issue includes two neuroimaging studies. Zhang et al. (Contribution 4) examined hippocampal macrostructural and microstructural alterations using MRI with structural and diffusion kurtosis imaging techniques in the AD continuum. During the AD dementia stage, the best discriminator is the macrostructural index (i.e., right hippocampal volume). In contrast, in the predementia stage of SCD and MCI, the best indicators are microstructural measurements (i.e., left hippocampal mean kurtosis for SCD and right hippocampal mean diffusivity for MCI). Accordingly, these imaging techniques may represent a new approach for determining early-stage SCD. Takahashi et al. (Contribution 5) used NIRS to measure brain oxyhemoglobin signals during category fluency, finger tapping, and dural tasks in older adults with and without MCI. The discrimination accuracy of older adults with and without MCI was low; however, the signal changes differed from those of younger adults. Therefore, these tasks may be useful for detecting functional changes in the prefrontal cortex of elderly adults with and without MCI.

Research on body fluid biomarkers for early AD detection is underway. Typical biomarkers include amyloid and tau proteins [11], which are included in the National Institute on Aging–Alzheimer’s Association Research Framework standards for understanding the pathology of AD [12]. In this regard, Zhang et al. (Contribution 6) reported that YKL-40 in cerebrospinal fluid is useful for early AD screening, which is closely related to neuroinflammation, based on a systematic review and meta-analysis. In addition, YKL-40 in the plasma could distinguish between patients with AD and healthy individuals, indicating its usefulness for early AD screening and monitoring.

### 2.2. Early Intervention in the AD Continuum

The following 12 modifiable risk factors for dementia have been identified: a low level of education, hearing loss, traumatic brain injury, hypertension, alcohol consumption, obesity, smoking, depression, social isolation, physical inactivity, air pollution, and diabetes. Up to 40% of dementia cases can be prevented or delayed by modifying these risk factors [13]. Meanwhile, World Health Organization guidelines recommend several measures to reduce the risk of cognitive decline, including physical activity, tobacco cessation, nutritional factors, alcohol use disorder, cognitive interventions, social activity, weight, hypertension, diabetes mellitus, dyslipidemia, depression, and hearing loss management [14].

From the perspective of interventions for physical inactivity, Yamasaki (Contribution 7) reviewed the effects of physical activity and exercise-related interventions in older adults with and without cognitive impairment and summarized their possible mechanisms. Notably, although physical activity is known to be divided into open-skill and closed-skill exercises [15], the author has demonstrated that open-skill exercises have a higher protective effect on cognitive function than closed-skill exercises. Consequently, the need to actively promote open-skill exercise interventions has been emphasized.

Nath et al. (Contribution 8) explored the cognitive and biomarker outcomes for patients with MCI and healthy elderly adults after a single session of an interactive physical and mental exercise intervention (20 min of pedal-to-play exercise). The results showed that patients with MCI showed improved executive function and greater increments in alpha-amylase levels (related to neurogenesis) compared with healthy older adults, suggesting the usefulness of neuro-exergame for them.

Pozzi et al. (Contribution 9) reviewed the most important advances in the prevention of cognitive impairment in elderly adults and provided an overview of future steps in this field. In particular, the authors highlight the usefulness of Brain Health Services for people with SCD, which includes risk assessment, risk communication, and tailored interventions to reduce the risk of dementia. The authors emphasized the need for global collaboration and intensified research efforts to address the intricate determinants of brain health and their potential impact on healthcare systems worldwide.

### 2.3. Other Types of Dementia and High-Risk Conditions of Dementia

This Special Issue also includes papers on major types of dementia other than AD. Criteria for prodromal DLB, including clinical features and biomarkers, have recently been proposed [16]. Phillips et al. (Contribution 10) investigated the sensitivity of this diagnostic criterion. Of the thirteen patients who met the diagnostic criteria for prodromal DLB, only one patient progressed to DLB after two years. Based on these findings, the authors argued that more stringent diagnostic criteria are needed to stratify the risk of developing DLB more accurately.

Cognitive decline is a common complication of stroke [17]. A meta-analysis conducted by Wang et al. (Contribution 11) investigated the relationship between acute ischemic stroke-related C-reactive protein (CRP) levels and post-stroke cognitive decline. An analysis of nine cohort studies involving 3893 patients with stroke revealed that patients with high CRP levels during the acute phase of ischemic stroke may be at a higher risk of cognitive decline. Thus, CRP may be a biomarker of cognitive decline after stroke.

One of the major risk factors for dementia is a condition called MCR, which is defined by slow gait and the presence of SCD [6,18]. Merchant et al. (Contribution 12) investigated the association between MCR and body composition, including sarcopenia and systemic inflammation in pre-frail older adults. They found that MCR was associated with sarcopenia and systemic inflammatory biomarkers (interleukin-10 [IL-10] and IL-10/tumor necrosis factor-α). The authors state that this study is one of the first to demonstrate an association between IL-10 and MCR in pre-frail older adults and could serve as a future therapeutic target.

Motor signs and cognitive performance appear to be parallel manifestations of underlying brain disease. Siokas et al. (Contribution 13) examined the association between motor signs and cognitive performance in older adults without cognitive impairment. The results showed that people who had trouble getting up from a chair had worse episodic memory, semantic memory, processing speed, and executive function. In contrast, those who were slow to move had worse language, processing speed, and executive function. Consequently, the authors stated that difficulty in rising from a chair and bradykinesia are clinical indicators of cognitive decline in the elderly.

Sleep deprivation, especially rapid eye movement (REM) sleep deprivation, can affect mood, learning, and memory function and can even lead to the development of neuropsychiatric disorders, including AD. Using REM sleep-deprived rats, Liu et al. (Contribution 14) reported that Gastrodin (the active ingredient in *Gastrodia elata*) treatment significantly improved sleep disturbance, cognitive impairment, and neuronal damage in the hippocampal CA1 region and cerebral cortex. Accordingly, Gastrodin is a potential candidate for the treatment of REM sleep deprivation.

## 3. Conclusions

This Special Issue summarizes research on brain health maintenance, especially early detection and intervention in dementia and high-risk conditions. We hope that this Special Issue will be useful in clinical practice and will provide suggestions for future research.
